# Supplementary material for: Assessment of awareness and attitudes of pregnant women toward labor epidural analgesia and factors influencing the decision to opt for it: A cross-sectional study
Source: PLoS One. 2025 Nov 26;20(11):e0317476. doi: 10.1371/journal.pone.0317476 (PMC12654905; doi:10.1371/journal.pone.0317476)
Supplement: Attachment — Submitted filename: Response to Reviewers.docx [file pone.0317476.s005.docx]

**Response to reviewers**

Dear Academic editors and Reviewers,
Thank you very much for your invaluable comments on my manuscript. Your insightful feedback has significantly improved the quality of my paper. Below is my response table addressing each point raised by the editors and reviewers.

|  | **Editor’s comment** | **Response** |
| --- | --- | --- |
| 1 | Please ensure that your manuscript meets PLOS ONE's style requirements, including those for file naming | The manuscript revised according to PLOS ONE formatting style and the following changes are made:   - The title, author names, affiliations, and the main body were revised according to the PLOS ONE formatting requirements |
| 2 | Please provide additional details regarding participant consent: (a) whether consent was informed and (b) what type you obtained. | Details included as suggested. |
| 3 | In the online submission form, you indicated that Data can be made available to researchers upon request by contacting the authors.  All PLOS journals now require all data underlying the findings described in their manuscript to be freely available to other researchers. | Data uploaded as supplementary information. |
| 4 | Additional editor comments | All additional comments from the editor have been explicitly reviewed and incorporated. |
| 5 | Reviewer comments to the author | Noted and acknowledged. |
|  |  |  |
