# Supplementary material for: Assessment of awareness and attitudes of pregnant women toward labor epidural analgesia and factors influencing the decision to opt for it: A cross-sectional study
Source: PLoS One. 2025 Nov 26;20(11):e0317476. doi: 10.1371/journal.pone.0317476 (PMC12654905; doi:10.1371/journal.pone.0317476)
Supplement: Attachment — Submitted filename: Review report PLOS ONE.docx [file pone.0317476.s003.docx]

**General comment**

- This cross-sectional study aims to evaluate the awareness and attitudes of pregnant women regarding labor epidural analgesia, as well as the factors that influence their decision to choose this option (**Manuscript Number PONE-D-24-60257).**
- While the study's objective is clearly articulated and holds significant importance, the introduction, methods, and discussion sections require substantial enhancement.
- It is advisable for the authors to consult the STROBE guidelines for cross-sectional studies to ensure comprehensive reporting and to explicitly state adherence to these guidelines within the manuscript.
- Additionally, a thorough revision of the English language and proofreading is recommended, along with a major revision of the manuscript itself.

**In the abstract**

- It is essential to maintain proper spacing between text and punctuation throughout the manuscript. For example, in line 22, there should be a space after "countries." and before "Awareness," and in line 23, a space is needed before "Therefore."
- Furthermore, in line 25, the authors should specify the exact time frame of the study conducted from June to August 2024.
- It is also important to clarify the sampling method used
- The authors should indicate that as the study was conducted in the facility. Like a facility-based cross-sectional study design.
- In line 28, the phrase "multivariable logistic regression" should be revised to "Both bivariable and multivariable logistic regressions" to accurately reflect the analysis performed.
- Lastly, in line 20, the statement regarding epidural analgesia should be modified to specify "active first stage and second stage of labor," as the intensity of pain increases with cervical dilation beyond 4 cm, and the analgesia is most effective during this phase. It is crucial to note that administering epidural analgesia during the latent phase of labor may hinder labor progression.

**Keywords:** Labor Epidural Analgesia, Awareness and Attitudes, Decision-Making Factors, Pregnant Women.

- Please separate the awareness from attitude by using comma, and remove the word ‘and’

**Introduction**

- The introduction needs to be more focused on what the problem is
- The authors fail to clarify their criteria for selecting the literature they reference, which diminishes the credibility of their review.
- Furthermore, the literature review is notably limited, primarily drawing from studies within a single geographical region, rather than considering lessons from other parts of Africa or globally.
- It would enhance the introduction to include a discussion of various labor pain relief methods, both pharmacological and non-pharmacological, along with evidence supporting their effectiveness and implications for birth outcomes.
- Additionally, there is a lack of reference to the content in lines 61-63, which discusses the underutilization of labor epidural analgesia due to misconceptions and cultural beliefs that foster negative attitudes.
- The citation in reference number 6, mentioned in line 61, does not pertain to epidural analgesia and should be verified by the authors.
- Lastly, the introduction suffers from redundancy, particularly in the text found in lines 55-56, which could be streamlined for clarity.
- **The methodology section** of this study requires further elaboration, particularly regarding the specific procedures employed and the population densities in the areas under investigation.
- Additionally, the authors have not disclosed the number of anesthesiologists involved in the study, nor have they clarified whether the use of epidural analgesia during labor incurs any costs at their facility.
- There is also a lack of information regarding the total number of deliveries performed at the health facility and the qualifications of the personnel involved.
- Furthermore, the staff-to-patient ratios remain unspecified, and there is no mention of the training that providers receive, either pre-service or in-service, concerning epidural analgesia in labor.
- It is essential for the authors to distinguish between the source population and the study population, as well as to justify the exclusion of pregnant women in their first and second trimesters.
- The rationale behind the use of a correction formula for sample size calculation should also be addressed.
- Lastly, the dependent variables should be revised to focus solely on the "Awareness and attitudes of mothers toward labor epidural analgesia," omitting the reference to the decision to consider LEA during delivery.
- The authors did not provide details regarding their approach to engaging study participants, including the methods of invitation, the locations where interviews were conducted, and the average duration of these interviews for completing the questionnaire.
- It is essential to clarify the original source of the questionnaire, including the context in which it was first utilized, and whether it underwent validity and reliability testing at that time.
- Additionally, if any modifications were made to the tool, an explanation for these changes should be included.
- The questionnaire used should be described, referred to within the manuscript, and made available as an appendix
- The methods section ought to outline the structure of the tool, detailing the number of sections, the content and purpose of each section, and the number of items or questions per section. This information will enhance the reader's understanding of how the tool facilitated the researchers in addressing the research questions and assessing outcomes.
- Furthermore, incorporating a conceptual framework that illustrates the anticipated relationships among the study variables would provide clarity on the variables adjusted for in the multivariable logistic regression model.
- Lastly, it is important to describe the background of the data collectors, such as their qualifications as health professionals.

**Result**

- In table 1, the criteria used to classify study participants into income brackets, such as <10,000 ETB and 10,000-25,000 ETB, should be clearly defined.
- In Table 2, the phrase "Decision to take LEA (Yes/No/Not sure)" lacks clarity and requires further explanation to ensure that readers fully understand the context and implications of these responses.

**Discussion**

- This section requires significant improvement to effectively illustrate the connections among various components of the research paper and to highlight the significance of the findings.
- The authors have not sufficiently analyzed, explained, or interpreted their results; instead, they have largely reiterated the content from the results section and merely compared their findings to previous studies.
- A concise summary of the key findings should be presented at the beginning, contextualized within the existing literature.
- Furthermore, the recommendations appear more suited for a report than a scientific publication, often lacking clarity due to missing words.
- The authors fail to specify who should implement the suggested actions or how these actions would be executed.

**Limitations**

- The exclusion of mothers in labor represents a notable limitation of this study, as it may significantly affect the assessment of the need for labor pain relief.

Thank you!
