# Supplementary material for: Assessment of awareness and attitudes of pregnant women toward labor epidural analgesia and factors influencing the decision to opt for it: A cross-sectional study
Source: PLoS One. 2025 Nov 26;20(11):e0317476. doi: 10.1371/journal.pone.0317476 (PMC12654905; doi:10.1371/journal.pone.0317476)
Supplement: Attachment — Submitted filename: comments.docx [file pone.0317476.s004.docx]

Dear Editor,

I appreciate the invitation to discuss the intriguing title, "Assessment of Awareness and Attitudes of Pregnant Women toward Labor Epidural Analgesia and Factors Influencing the Decision to opt for it: A Cross-Sectional Study." This study promises to offer valuable insights for enhancing obstetric care, management, and outcomes.

The manuscript is well-written and easy to understand. I appreciate the authors for their effort in producing this work.

I have some comments and suggestions, which you can find below.

General suggestions

- Adhere with the journal guidelines.
- Please revise the introduction section, as it appears fragmented. Additionally, some sentences lack references. Acknowledge the sources, or if they are from similar sources, use conjunctions to combine them. Some paragraphs feel disjointed. Use transitional phrases like “However,” “In contrast,” or “Furthermore” to create flow.
- Try to address punctuation errors throughout the document.
- Make the references uniform example reference 17

**Introduction section:-**

-Avoid generalizations such as "worst pain ever." Therefore, modify lines 44-45 to read: "While childbirth is often anticipated as a joyful milestone, it is also associated with significant pain, which can be one of the most intense experiences in a woman’s life."

- I do not see the importance of the sentence mentioned by the authors in lines 52-54, particularly regarding your study's aim. It would be better for the authors to omit it.

- The authors stated in lines 64-65 that "there are no published studies in Ethiopia investigating the awareness and attitudes of pregnant women toward LEA or assessing the factors contributing to its poor utilization." However, reference 10, conducted by Workie MM et al. in 2021, presents a similar study. Therefore, please revise this justification section.

- I recommend that the authors revise the justification of the study, as it lacks coherence and contains redundant phrases, such as "the aim of this study," in multiple paragraphs (e.g., lines 68-70 and 81-83). Additionally, it should be made more concise while maintaining the flow of ideas and improving the overall presentation.

**Method section**

-The authors should format the inclusion criteria as a heading to ensure consistency with the other headings.

- Why did the authors exclude patients with a previous history of epidural analgesics? I recommend that they consider this factor when determining the outcome. The reasons included in lines 101-102 do not seem particularly necessary. Moreover, if the questionnaire was self-administered, please note that individuals who cannot read or are not educated were excluded.

- Sample size determination section: If there were 880 individuals expected to attend ANC follow-up, why did the authors not use the calculated sample size of 384 instead of applying the population reduction formula?

- Sampling technique: Was the list of 880 individuals accessible for systematic random sampling? I am concerned that not all expected individuals may attend the full ANC follow-up during the third trimester of pregnancy for various reasons.

-Data analysis: The authors used the phrase "bivariate analysis" in lines 131 and 133, which is not appropriate in the context of this study. This term implies a relationship between **two** predictors, rather than between a **predictor and** an outcome. Therefore, it should be changed to "bivariable analysis." Please address similar errors throughout the manuscript example there is similar error in line number 174.

- Ethical considerations: The authors need to include the fact that the study followed the Helsinki guidelines for human research in this section.

**Result section**

-In line 149, please insert a full stop before the word “Therefore.” Additionally, in line 151, omit the use of the double hyphen in “25--34 years age group.”

- In line 175, the phrase “Perception of LEA causing significant problems for the mother and fetus” needs modification, as the perceived problems for the mother and fetus are distinct contributing factors. Please split it into two separate statements.

-In the tables, please ensure they are self-explanatory regarding why, where, and when. The authors missed the year of study.

- In "Table 2," under the variable "Education," the authors listed the p-value for the variable they considered constant. This may be a typographical error, so please correct it. Additionally, please remove the merging of the table in the boxes indicating the p-value to reduce ambiguity for readers.

**Discussion section**

- The authors were certain that the causes of the unwillingness to accept LEA include perceived interference with the labor process and the additional costs associated with the technique. However, these factors were not studied, so where did you obtain these reasons?

- In line 194, the authors mention “the relatively high level of awareness observed in this.” What does this relatively high awareness mean? Compared to what? Is there any standard for this assessment?

- Overall, in the discussion section, the authors should refrain from hastily generalizing their findings in relation to previous studies, such as those from urban research. In low-income countries, where awareness levels are often low, it is essential to include specific numeric figures, such as the percentage of awareness, along with appropriate citations for each statistic. Additionally, they should ensure that the percentages from previous studies fall within the confidence interval of the current study's outcome values to say consistent with the current study. Similarly, in lines 218-220, the authors state that “Multiple other studies conducted in different parts of the world have also identified the level of education as an important factor influencing pregnant women’s decision to request LEA.” Please specify the countries where these studies were conducted, along with the respective citations, rather than citing them collectively. This suggestion also applies to other contributing factors.

**Conclusion section**

- The authors conclude that in lines 259-260, “This reluctance appears to stem from inadequate explanations provided by healthcare professionals, potentially leading to negative perceptions.” However, these variables were not associated with your outcome, so please base your conclusions on your findings.
