# Supplementary material for: Assessment of awareness and attitudes of pregnant women toward labor epidural analgesia and factors influencing the decision to opt for it: A cross-sectional study
Source: PLoS One. 2025 Nov 26;20(11):e0317476. doi: 10.1371/journal.pone.0317476 (PMC12654905; doi:10.1371/journal.pone.0317476)
Supplement: Attachment — Submitted filename: Response_to_Reviewers_auresp_2.docx [file pone.0317476.s006.docx]

**Response to reviewers**

Dear editors and reviewers, thank you very much for your insightful and thorough feedback, which has greatly improved our manuscript. We sincerely appreciate the time and expertise you have invested in reviewing our work.

|  | **Review #2’s comment** | **Response** |
| --- | --- | --- |
| 1 | the introduction, methods, and discussion sections require substantial enhancement. | Revised as suggested |
| 2 | It is advisable for the authors to consult the STROBE guidelines for cross-sectional studies to ensure comprehensive reporting and to explicitly state adherence to these guidelines within the manuscript. | Improvised all parts of the manuscript based on the STROBE guideline |
| 3 | A thorough revision of the English language and proofreading is recommended, along with a major revision of the manuscript itself. | Revised as suggested |
| 4 | The citation in reference number 6, mentioned in line 61, does not pertain to epidural analgesia and should be verified by the authors. | The mentioned study reported that the labor analgesia utilization, including epidural, is low in Ethiopia |
|  | The authors have not disclosed the number of anesthesiologists involved in the study, nor have they clarified whether the use of epidural analgesia during labor incurs any costs at their facility | This study aimed to evaluate pregnant women’s awareness and attitudes toward labor epidurals and identify factors influencing their decision to use epidural analgesia. Since these factors were thoroughly examined in a prior study assessing LEA satisfaction at this facility (URL: <https://www.emjema.org/index.php/EMJ/article/view/2741>), they were not considered relevant to this study. |
|  | It is essential for the authors to distinguish between the source population and the study population, as well as to justify the exclusion of pregnant women in their first and second trimesters. | Source population added and also justification for including only 3^rd^ trimester pregnant women added. |
|  | The rationale behind the use of a correction formula for sample size calculation should also be addressed. | Justification added. |
|  | The authors did not provide details regarding their approach to engaging study participants, including the methods of invitation, the locations where interviews were conducted, and the average duration of these interviews for completing the questionnaire. | All these details included in the data collection procedure part. |
|  | It is essential to clarify the original source of the questionnaire, including the context in which it was first utilized, and whether it underwent validity and reliability testing at that time. | Details included and all other suggestions in the methods part addressed as much as possible. |
|  | On Results: In table 1, the criteria used to classify study participants into income brackets, such as <10,000 ETB and 10,000-25,000 ETB, should be clearly defined. | By the time of data collection, 10000 ETB was equivalent to $100 USD. Therefore, the classification was to assume <$100, $100-$500, and >$500 to classify as low income, middle income and high income families. |
|  | Comments on Result and discussion parts | Suggestions on results and discussion part addressed as much as possible |
|  | Author # 2 |  |
|  | The authors stated in lines 64-65 that "there are no published studies in Ethiopia investigating the awareness and attitudes of pregnant women toward LEA or assessing the factors contributing to its poor utilization." However, reference 10, conducted by Workie MM et al. in 2021, presents a similar study | Noted and revised. |
|  | - Sampling technique: Was the list of 880 individuals accessible for systematic random sampling? I am concerned that not all expected individuals may attend the full ANC follow-up during the third trimester of pregnancy for various reasons. | We used systemic sampling technique based on the predicted flow of 3^rd^ trimester pregnant women visiting the center in the data collection period. |
|  | The authors were certain that the causes of the unwillingness to accept LEA include perceived interference with the labor process and the additional costs associated with the technique. However, these factors were not studied, so where did you obtain these reasons? | Revised accordingly. |
|  | In line 194, the authors mention “the relatively high level of awareness observed in this.” What does this relatively high awareness mean? Compared to what? Is there any standard for this assessment? | The authors mean relative to 2 studies conducted in Ethiopia, and a couple others from Africa. |
|  | All other comments and suggestions | Addressed as much as possible. |
