# Supplementary material for: Assessment of awareness and attitudes of pregnant women toward labor epidural analgesia and factors influencing the decision to opt for it: A cross-sectional study
Source: PLoS One. 2025 Nov 26;20(11):e0317476. doi: 10.1371/journal.pone.0317476 (PMC12654905; doi:10.1371/journal.pone.0317476)
Supplement: Attachment — Submitted filename: Response to Reviewer Oct 2025.docx [file pone.0317476.s007.docx]

**Response to reviewer**

|  | **Editor** |  |
| --- | --- | --- |
|  | The abstract need to be presented with subtitle of background, methods, results and conclusion per the journal standard. | Done. |
|  | Please avoid bullet point at the end of introduction | Done |
|  | The document still needs language improvement, typos and grammatical correction throughout the entire document. | Revised thoroughly. |
|  | Please revise the inclusion criteria | Revised as suggested. |
|  | Please refrain from including details immediately after the subtitle; instead, place all information below it. | Revised accordingly. |
|  | Please ensure that the font size and theme fonts are applied consistently throughout the entire document. | Revised and edited. |
|  | Please provide details on how the data were transitioned from bivariate to multivariate analysis, as well as how multicollinearity was assessed in the data analysis section. | All suggested details included! |
|  | Please correct the punctuation and grammar in the results sections. | Done. |
|  | Please verify the frequency and percentage in Table 1 against the total sample size. For example, the total for the age group category is 167, while the total frequency for the educational level is 230. | This discrepancy is attributed to missing data, as 63 participants did not provide their age on the questionnaire. |
|  | The strengths of your study should be highlighted in the "Strengths and Limitations" section at the end of the discussion | Included. |

Dear Academic Editor and Reviewers,
I sincerely appreciate your thoughtful feedback and constructive suggestions. Your input has greatly improved the quality of this manuscript and contributed to my growth as a researcher. Thank you very much for your valuable support.
